# Polio Travel Restrictions: A Sledgehammer to Crack a Nut?

**DOI:** 10.12669/pjms.304.5810

**Published:** 2014

**Authors:** Rashid Jooma

**Keywords:** Polio, Eradication, Pakistan, Mandatory vaccination

## Abstract

Imposition by WHO of mandatory vaccination for international travelers from Pakistan has caused confusion and panic amongst travelers besides the adverse effect on the country’s image from the widely reported statement. It is felt that the announced measure is not primarily supported by science but is rather a response to disillusionment in the donors about the repeatedly missed eradication targets set by WHO. In the past few years, exportation of poliovirus from Pakistan has caused outbreaks in China, Iraq and Syria besides the ongoing two way transmission with Afghanistan, but the carriers in these spreads are mainly land route travelers. Vaccinating all air travelers is unnecessarily punitive besides being probably ineffectual in halting exportation.

The unrelenting focus on polio eradication may have negative impact on fragile health systems besides arousing suspicions of religious elements opposed to vaccination. Routine vaccination and polio campaigns as part of community development measures such as provision of clean drinking water and elimination of surface sewage drainage would be more accepted. The government would be well advised to assume control of the polio eradication program and make it a national development issue.

There are currently 3 countries where poliomyelitis is endemic, yet of them, it was Pakistan that was recently singled out for imposition of mandatory requirements of vaccination on international travellers.^1^ Within the country there has been considerable consternation, besides embarrassment, on the travel restrictions and it is felt that the measure was not primarily supported by science but rather was grandstanding by WHO, to convey intensity of purpose to their donors and the global health community at large. In a speech celebrating the elimination of polio in India, the Director General of WHO, in November 2012, stressed the “need to keep the pressure on governments” of the remaining endemic countries.^2^

Portraying the recent intensification of polio virus circulation in the unvaccinated agencies of the Federally Administered Tribal Area (FATA) and exportation of virus as “an extraordinary event”, the WHO convened its Emergency Committee under the International Health Regulation 2007 for only the 3^rd^ time to declare a Public Health Emergency of International Concern (PHEIC).^3^ The first declaration of a PHEIC was on the occasion of the H1N1 pandemic in 2009 which as we now know, turned out to be a false alarm causing international panic and huge economic losses to the travel industry and unnecessary diversion of health sector budgets to stockpiling of supplies in countries, many of which could ill afford the unplanned expenditure.^4^ Similarly, Pakistan can now expect to bear the losses from the disruptions of international travel and the discouragement of commercial travelers from visiting the country besides the adverse effect on the nation’s standing consequent to the highly publicized WHO strictures.

The WHO statement mandating polio vaccination of all travelers links Pakistan to ongoing exportation of poliovirus to Afghanistan in the current year;^1^ in previous years, outbreaks in China, Syria and Iraq have been identified on genetic sequencing as being of Pakistani origin. However, it is the land route traveler that is mainly involved in these exportations particularly to adjacent Afghanistan. There are over 50,000 passengers leaving the airports of Pakistan for foreign destinations each week and documented carriage of virus by air travelers are few and far between.^5^ The measures imposed seem to be akin to use of a sledgehammer to crack a nut and the nation is being made to suffer collective punishment for the sins of a misguided minority.

In the context of the repeated setting of target dates for polio eradication since 1988, it does seem that the Global Polio Eradication Initiative (GPEI) has its credibility challenged and in seeking the Holy Grail, WHO is losing sight of its fundamental objective: fullest attainment of health. Polio vaccination has become an end unto itself irrespective of its effects on a fragile health system. And it is this unrelenting focus on polio that arouses the suspicions of the naysayers. Extending similar effort and resources into other interventions such as improving water and sanitation could mitigate these suspicions. The open sewers of the urban slums and the villages and towns of the tribal agencies where most polio cases are occurring have a multiplier effect on polio virus circulation in children without robust routine immunization.^6^ A program in FATA and high-risk districts of Karachi, based on community participation, to replace the surface drainage of domestic effluent with submerged sewage pipes separated from the water supply, would have health benefits impacting positively on polio control and bring goodwill. Provision of vaccinations in Pakistan would certainly have greater uptake as part of more broad-based community development programs linked to strengthened routine immunization.^7^

What is being overlooked is the notable progress that has been made in Pakistan by the polio eradication program. There was a decline in the incidence of paralytic poliomyelitis from 1155 cases in 1997 to 28 in 2005 when negative propaganda under the guise of religion increased the number of refusals in polio campaigns amongst the Pashtun populations. This trend has been most evident in the conflict zone of FATA where polio is intensifying in more recent times even though decreasing in the rest of the country.^8^ There has been a rundown of polio cases to zero in the seemingly intractable border districts of Baluchistan and no case has been reported so far this year from the largest province of Punjab where immunity now seems robust. The nidus of polio in Pakistan is now restricted to the agencies of FATA that have been excluded from vaccination for the past 2 years and is spread by migration to regional and distant districts (and transnationally) from this reservoir of virus circulation.^8^ The ongoing civil war that denies vaccination to the resident children by the Taliban is the barrier that will have to be overcome to eradicate polio. At present that does not seem feasible and the increasing influence of Islamic militant forces in Afghanistan and Iraq, despite the best efforts of the USA and its NATO allies, does not give cause for optimism. In the eyes of the Taliban, preventing polio vaccination in their areas of control hurts the interests of the western powers and what they view as the client government of the west in Islamabad and for them, preventing access for immunization has become an instrument of war. Until the writ of the State is effectively asserted in the tribal agencies, by force of arms or by accommodation, it is clear that vaccination will be actively resisted and polio virus circulation will continue, making polio eradication unattainable.

But whichever path of conflict resolution the government of Pakistan chooses, it can create the conditions for polio eradication by taking responsibility for the program while guiding external donors to community development needs. Currently, the GPEI funds and technically implements the program with nominal leadership of the government. Amongst the health workers, the perception is that this is a WHO program and thus does not engender the same commitment as would for their indigenous responsibilities. Indeed many question if the district health leadership would really like to see polio eradicated and risk drying up of the financial incentives offered by the GPEI donors. What is clear is that the grass roots governance of the polio program in Pakistan is weak with low levels of interest and ownership. The upper echelons of political leadership are aligned with the eradication objectives but the district leadership does not seem to feel that polio vaccination has any bearing on their relationship with their political mentors. The WHO’s Independent Monitoring Board has recently castigated the Prime Minister’s polio monitoring cell and suggested that an Emergency Operations Center be set up with senior bureaucrats and politicians to energize the vaccination campaigns.^9^ But there has so far been no shortage of action plans, task forces and committees basking in the perks and privileges allowed by GPEI’s generosity. Clearly, accountability cannot be properly mandated through a committee and for the state health worker, is at its best when enforced by statutory responsibility and buttressed by adequate remunerations.

A recently published survey of 946 university students in Pakistan points to the widespread ambivalent attitudes to the polio eradication program in the country. Though the overwhelming majority understood that polio was a major public health problem confronting the nation and that vaccination was efficacious, almost a third held the opinion that all those working for the program including UN staff were spies working for western governments or their agencies while the same proportion replied either yes or don’t know to the question “is killing of polio vaccinators justified?”^10^ If this is the view of the educated elite, it is a disturbing indicator of the prevailing perceptions and must be countered for vaccination campaigns to regain traction.

In its current trajectory, Pakistan is on course to be the last place on earth with endemic polio unless it makes a radical departure from the present failing arrangements of the polio eradication program. It is proposed that there be an enhancement of the state’s commitment to public health with the requisite financial outlays being viewed as a critical facet of national development expenditures. The Government would be well advised to meaningfully assume leadership of the polio program with purchase of vaccine from their own resources, resuming executive control of immunization activities from the global polio partnership and paying the field health workers and vaccinators a living wage.^11^ The Indian Government took responsibility for vaccine purchase in 2010 leading to a greater national ownership and responsibility amongst the health workers with enhanced levels of immunization.^12^ This was the critical factor in their achieving eradication soon thereafter and could be for Pakistan as well.
